# Nationwide trends in psychiatric prescription practices for drugs with putative cognitive adverse effects in schizophrenia spectrum and bipolar disorders from 2013 to 2022

**DOI:** 10.1080/20523211.2026.2650543

**Published:** 2026-03-31

**Authors:** Nathan Vidal, Solène Frileux, Nadia Younès, Emmanuelle Corruble, Christine Passerieux, Eric Brunet-Gouet, Paul Roux

**Affiliations:** a Centre Hospitalier de Versailles, Service Universitaire de Psychiatrie d’Adultes et d’Addictologie, Le Chesnay, France; b DisAP, MOODS team, INSERM UMR1018, CESP, Université de Versailles Saint-Quentin-En-Yvelines – Université Paris-Saclay, Le Chesnay, France; c Université Paris-Saclay; Université de Versailles Saint-Quentin-En-Yvelines; DevPsy-CESP, INSERM UMR1018, Villejuif, France; d MOODS team, INSERM UMR1018, CESP, Faculté de Médecine Paris-Saclay, Université de Versailles Saint-Quentin-En-Yvelines, Université Paris-Saclay, Villejuif, France

**Keywords:** Psychotropic drugs, bipolar disorders, schizophrenia, cognition, anticholinergic drugs

## Abstract

**Background:**

The management of schizophrenia spectrum (SZ) and bipolar disorders (BD) can involve psychotropic polypharmacy, high psychotropic doses, a high anticholinergic burden, and the use of anticholinergic agents and benzodiazepines, which are associated with poorer cognitive performance. We evaluated trends in those prescription practices in adult outpatients with SZ or BD between 2013 and 2022, to guide future treatment guidelines and interventions for improving cognition.

**Methods:**

We performed a retrospective longitudinal analysis of the nationwide French health claims database. We identified the deliveries of psychotropic drugs (antipsychotics, antidepressants, anxiolytics, hypnotics, and antiepileptics) occurring between 2013 and 2022. We estimated mixed-effects linear regression models of the number of psychotropics prescribed, the total daily dose/DDD of psychotropics, cumulative anticholinergic burden, and the frequencies of benzodiazepine and anticholinergic agent use across diagnoses and age groups.

**Results:**

Between 2013 and 2022, we measured small but significant declines in the number (*β* between −0.006 and −0.031) and dose of psychotropic drugs (*β* between −0.003 and −0.029) and the frequency of benzodiazepine use (*β* between −0.26% and −0.88%) within most groups. The use of anticholinergic agents decreased in adults with SZ but not BD, and the anticholinergic burden remained globally constant. In 2022, 42.1% adults with BD and 49.4% with SZD had at least once a high anticholinergic burden due to psychotropic drugs. Most trends towards deprescription halted after 2020.

**Conclusion:**

The slight decrease in the number and total dose of psychotropic drugs and the deliveries of benzodiazepines from 2013 to 2022 suggest a better consideration of adverse effects in adults with SZ or BD. However, the use of anticholinergic agents and the anticholinergic burden did not consistently decrease, suggesting that prescribers fail to reduce anticholinergic burden. Efforts to support deprescribing are further required after the pandemic.

## Background

The management of schizophrenia spectrum (SZ) and bipolar disorders (BD) typically requires the use of psychotropic drugs to treat and prevent acute episodes in inpatient and outpatient care (Huhn et al., 2019; Yatham et al., 2018). Outside of acute episodes, practices such as psychotropic polypharmacy, the use of high doses, anticholinergic agents and benzodiazepines, and exposure to a high anticholinergic burden – the cumulative anticholinergic properties of the medications – are associated with additional cognitive impairment in patients with SZ (Ballesteros et al., 2018; Chakos et al., 2006; Georgiou et al., 2021; Mancini et al., 2025; Savić et al., 2021; Vidal et al., 2024) and BD (Dias et al., 2012; Jamrozinski et al., 2009; Savić et al., 2021; Vidal et al., 2023). Impaired cognition may lead to poorer psychosocial functioning (Ehrminger et al., 2021; Kharawala et al., 2022) and more symptoms (Ehrminger et al., 2022; Martínez-Arán et al., 2004). The cognitive performance of individuals with SZ (Joshi et al., 2019; Kitajima et al., 2012; Lupu et al., 2021; Singh et al., 2022) and BD (Baandrup et al., 2017; Dias et al., 2012; Lupu et al., 2021) can be improved by reducing the dose of antipsychotics or anticholinergic burden, or deprescribing anticholinergic agents or benzodiazepines. Some evidence even suggests the reduction of anticholinergic burden could reduce the risk of dementia in adults with BD or SZD (Coupland et al., 2019; Gildengers et al., 2023). These changes in practice were recommended in recent guidelines (National Institute for Health and Clinical Excellence., 2014; Yatham et al., 2018), but there is a time lag between the formulation and implementation of recommendations (Morris et al., 2011). It therefore remains unclear whether the prescription of drugs with potential cognitive adverse effects has really decreased in recent years in patients with SZ and BD. This description of prescribing trends may help to guide the development of future treatment guidelines and interventions for improving cognition.

The prevalence of psychotropic polypharmacy – the use of multiple psychotropic drugs – increased from 20% in 2004 to 32.9% in 2012 among people with SZ in the UK (Heald et al., 2017). However, the use of multiple antipsychotics in SZ patients has decreased over the last two decades in various countries (Heald et al., 2017; Ying et al., 2023). The increase in psychotropic polypharmacy can be attributed to the 15–17% increase in mood stabiliser and antidepressant prescriptions in SZ patients over this period (Ying et al., 2023). No study assessing changes in the number of psychotropic drugs prescribed for SZ patients since 2012 has been published, to our knowledge, despite the association between polypharmacy and impaired executive function and reasoning (Vidal et al., 2024). Psychotropic polypharmacy is common in BD as it is recommended when treatment is insufficient to stabilise the patient: over 80% of people with BD are on more than two psychotropic medications (Fornaro et al., 2016). Between 2006 and 2019, the rate of patients with two psychotropics remained stable at hospital discharge, whereas the rates using three or more mood stabilisers and antipsychotics increased from 3.3% to 11.9% in Taiwan (Chiu et al., 2024). This finding probably reflects an increase in the use of antipsychotic drugs in BD treatment over the last decade (Doane et al., 2021). Psychotropic polypharmacy in BD is associated with detrimental effects on motor speed, verbal memory and other cognitive domains, particularly when antipsychotics are involved (Dias et al., 2012). Finally, the COVID-19 pandemic was associated with an increase in the number of psychotropic drug deliveries in the French general population (Benistand et al., 2022), probably contributing to the increase in psychotropic polypharmacy rates in patients with BD and SZ.

The daily doses of antipsychotic agents, antidepressants and antiepileptics prescribed for SZ increased between 1996 and 2005, whereas the doses of anticholinergic agents, benzodiazepines and lithium remained stable, resulting in an increase in the total dose of psychotropic drugs over this period (Nielsen et al., 2010). A higher daily antipsychotic drug dose is associated with poorer global cognition in first-episode psychosis (Ballesteros et al., 2018), and the reduction of antipsychotic dose in stabilised SZ patients improves neurocognitive function, including processing speed in particular, without increasing the risk of relapse (Singh et al., 2022). In BD, antipsychotics were generally prescribed at the lowest end of the recommended dose range worldwide between 1998 and 2014 (Doane et al., 2021) and the mean doses of lithium, carbamazepine, and valproate remained unchanged from 1998 to 2001 in South Korea (Bahk et al., 2004). Few studies have investigated dosing trends for psychotropic drugs in BD, despite the known existence of links between higher antipsychotic dosage, mood stabiliser dosage and poorer cognitive performance (Jamrozinski et al., 2009; Steen et al., 2016). Investigations of recent trends in the number and doses of psychotropic drugs in SZ and BD outpatients, particularly since the end of the COVID-19 pandemic, could help to identify future challenges for reducing iatrogenic cognitive impairment.

In SZ, anticholinergic agents are usually prescribed to prevent antipsychotic drug-induced extrapyramidal side effects, the prevalence of which is highly heterogeneous, ranging from 9 to 63.7% across countries (Ying et al., 2023). The prevalence of anticholinergic agent use in outpatients with SZ declined in Taiwan, Denmark, and Austria between the 1990s and early 2000s (Ying et al., 2023). In European hospitals, the use of these drugs fell from 19.2% in 2000 to 11.4% in 2015 (Toto et al., 2019). However, data on outpatient trends after 2006 are lacking. In BD, only 2% of patients are treated with anticholinergic agents (Vidal et al., 2023), but the recent increase of the prevalence of antipsychotics may have led to an increase in the use of anticholinergic agents. Other psychotropic drugs also contribute to the anticholinergic burden. The prevalence of high anticholinergic burden varies greatly: 26.7–78.9% among clinically stable patients with SZD, and 21.2–67% among those with BD, depending on the scale used (Eum et al., 2017; Vidal et al., 2023, 2024). These values are significantly higher than the prevalence of 31.5–38.3% reported for the general population (Reinold et al., 2021). No study to date has evaluated the trends in anticholinergic burden over time in SZ and BD patients, despite evidence suggesting that cognition can be improved by tapering or deprescribe anticholinergic medications (Joshi et al., 2019; Mancini et al., 2025). However, no randomised intervention on anticholinergic burden has been published so far, making current evidence exclusively correlational.

Finally, 28.1% of people with SZ in Europe were prescribed a benzodiazepine between 2000 and 2005 (Haro et al., 2006). The prevalence of benzodiazepine prescription of inpatients with SZ increased between 2000 (29.3%) and 2015 (35.6%) (Toto et al., 2019). The use of benzodiazepines by outpatients with BD decreased significantly, from 51% in 2001 to 36% in 2012, in Denmark (Bjørklund et al., 2016), mirroring trends observed in the French general population over the same period (Bénard-Laribière et al., 2017). However, in the French general population, the use of benzodiazepines increased after the COVID-19 pandemic (De Bandt et al., 2024). Assessments of recent trends in benzodiazepine use post-pandemic might reveal a possible change in practices.

Overall, it remains unclear whether those prescription practices have effectively decreased in patients with SZD and BD over the past years. We aimed to describe their trends between 2013 and 2023 to guide prescribers’ initiatives to reduce iatrogenic cognitive impairment.

## Methods

### Participants

We conducted a nationwide non-interventional longitudinal retrospective analysis of French National Health Data System (SNDS) between January 1st, 2013, and December 31st, 2022. The SNDS includes records of medical interventions, hospital admissions and outpatient treatments for about 99% of the population (Bezin et al., 2017). Consent for this analysis was not required from the patients as the data are pseudonymised.

We included patients with schizophrenia spectrum (SZ) and/or bipolar disorders (BD) defined as those:

– Entitled to 100% reimbursement for long-term conditions within the year concerned for diagnoses corresponding to the following codes: F20-25, F28 or F29 for SZ, and F30-31 for BD, according to the international classification of diseases – 10th revision (ICD-10) (Supplemental Material Table S1)
– OR hospitalised at least once with a primary or related diagnosis of SZ or BD in the previous two years
– OR hospitalised at least once with a primary or related diagnosis of SZ or BD in the previous five years AND at least three deliveries of specific drugs in the year concerned: antipsychotic agents (ATC code beginning with N05A but excluding N05AN) for SZ, and antipsychotic drugs, lithium, valproate, carbamazepine, oxcarbazepine or lamotrigine for BD.

These criteria were based on the mapping of conditions, i.e. a list of consensual algorithms to detect patients with certain pathologies defined by the French National Health Insurance (Badjadj & Chan Chee, 2017; Chan Chee et al., 2017; Quantin et al., 2015, 2017). We excluded patients under 18 or over 65, to prevent mixing the different prescription patterns in these age groups. Twins and multiple births were excluded due to the impossibility of distinguishing between hospitalisations in the database. People with neurological diseases were censored at the first diagnosis of the disease, and people with mental retardation over the study period were excluded (see the definitions in Supplemental Material Table S2), as they may receive psychotropic medication for different reasons. For instance, anticholinergics can be prescribed for Parkinson's disease but not to counteract the adverse effects of antipsychotic drugs, their primary function in BD and SZ. We also excluded individuals who had undergone electroconvulsive therapy in the preceding year as such treatment can lead to a decrease in the prescription of psychotropic drugs, and those who were hospitalised monthly, as we analysed only outpatient prescriptions. Participants were included on diagnosis of BD and/or SZ and were removed from the cohort if no longer categorised as having SZ or BD, or when they reached the age of 66 years or died.

### Treatments

The participants’ reimbursement records were used to identify the prescriptions they had received. We collected data only for outpatient treatment delivery, which is exhaustive in the SNDS. If a person was hospitalised, information about treatment was considered missing. The duration of prescription was defined as the time between two treatment delivery dates. If no subsequent prescription was delivered within the maximum delivery period (Supplemental Material 1) or if no repeat delivery of the drug occurred before 31 December 2022, we considered the dose to be missing. For each patient and for each month, we measured:

– The number of psychotropic drugs, including antidepressants (ATC code N06A), anxiolytics (N05B), hypnotics (N05C, except N05CM, the code for phytotherapy), antipsychotics (N05A), and antiepileptics (N03).
– The total daily dose of psychotropic drugs. We measured the delivered dose in grammes for each treatment (dose per unit x number of units x number of boxes) and divided it by the prescription duration to determine the daily dose. Then, we calculated the ratio of the delivered daily dose to the defined daily dose (DDD), which is the mean maintenance daily dose of a drug used for its main purpose in adults determined by the World Health Organisation (WHO Collaborating Centre for Drug Statistics Methodology, 2013). The delivered daily dose/DDD ratio provides a standardised metric that facilitates comparisons across drug classes and studies (Lin et al., 2022; Nielsen et al., 2010). The DDD used was the one defined by the WHO as of May 2024. Finally, we summed these daily dose/DDD ratios to obtain the total dose of psychotropic drugs of the patient.
– The delivery of any anticholinergic agent (N04A).
– the cumulative anticholinergic burden of psychotropic drugs (including anticholinergic agents), calculated by summing the scores on Salahudeen's scale (Salahudeen et al., 2015), as recommended in SZ and in BD to assess central and cognitive anticholinergic adverse reactions (Vidal et al., 2023, 2024). We also conducted supplementary analyses including all medications in the anticholinergic burden, or using an alternative measure of cumulative anticholinergic burden, the CRIDECO Anticholinergic Load Scale (Ramos et al., 2022).
– The delivery of any benzodiazepine and related drugs (N05BA, N05CD, N05CF and N03AE).

### Demographics

We recorded sex at inclusion. The sample for each year was split into four age groups (18–34, 35–44, 45–54, and 55–65 years) to distinguish the changes in prescription practices over time and the changes in prescriptions resulting from patient aging.

### Statistics

Analyses were run in *R* software (version 4.3.1) and *SAS® Enterprise Guide* v7.4 (SAS Institute North Carolina, USA). We calculated the mean number and total daily dose/DDD for psychotropic drugs per patient, mean anticholinergic burden per patient, and the prevalence of benzodiazepine and anticholinergic agent deliveries in each month between 2013 and 2022, across three diagnosis groups (BD alone, SZ alone, and individuals belonging to both classes according to the algorithms) and the four age groups. Between 5 and 6% of the individuals included had a missing dose each month.

We used mixed-effects linear regression analyses to examine annual variations in these prescription practices across diagnoses and age groups from 2013 to 2022 to account for within-individual autocorrelation of repeated measures over time. The models included both random intercepts and random slopes for individuals. Time, in years, was included as a continuous variable, and the slopes obtained therefore represent the mean annual change in each variable. Analyses were conducted on complete cases. In additional analyses, we used segmented linear regression models to account for the impact of the COVID-19 pandemic (Supplemental Material 2).

## Results

### Description of the sample

**Figure 1.**
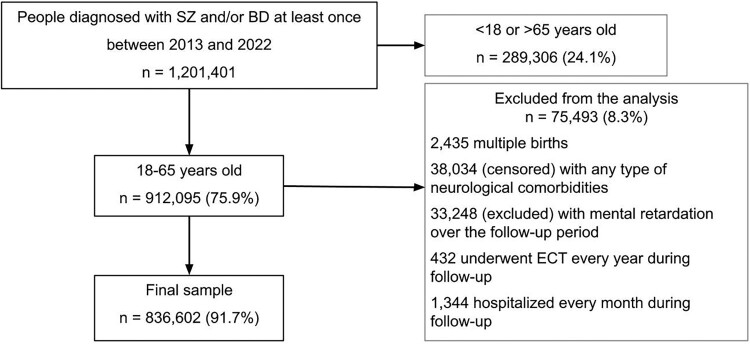
Flowchart of the study.

We identified 1,201,401 individuals with SZ or BD between 2013 and 2022 (Figure 1). The final sample consisted of 836,602 adults: 450,670 (53.9%) had SZ alone, 267,311 (32.0%) had BD alone, and 118,621 (14.2%) had both over the study period (Table 1).

**Table 1.** Description of the sample.

Characteristics		2013	2014	2015	2016	2017	2018	2019	2020	2021	2022	Total
N		434719	435229	434157	439524	446580	448725	448891	448783	450136	449955	836602
Female, n (%)		209892 (48.3%)	209281 (48.0%)	207906 (47.9%)	209657 (47.7%)	213504 (47.8%)	214148 (47.7%)	213787 (47.6%)	213012 (47.5%)	213531 (47.4%)	214080 (47.6%)	407237 (48.7%)
Age, mean (SD)		46.1 (11.5)	46.2 (11.5)	46.3 (11.5)	46.3 (11.5)	46.4 (11.6)	46.5 (11.6)	46.5 (11.7)	46.6 (11.7)	46.6 (11.8)	46.7 (11.8)	
Age group, n (%)	[18–34]	82458 (19.0%)	81458 (18.7%)	80042 (18.4%)	80448 (18.3%)	81218 (18.2%)	81766 (18.2%)	81822 (18.2%)	81862 (18.2%)	82977 (18.4%)	84204 (18.7%)	
	[35–44]	106045 (24.4%)	104900 (24.1%)	103548 (23.9%)	103275 (23.5%)	102318 (22.9%)	100937 (22.5%)	99687 (22.2%)	98820 (22.0%)	98233 (21.8%)	96928 (21.5%)	
	[45–54]	127098 (29.2%)	128616 (29.6%)	129703 (29.9%)	132532 (30.2%)	136121 (30.5%)	137092 (30.6%)	136274 (30.4%)	134752 (30.0%)	132909 (29.5%)	130862 (29.1%)	
	[55–65]	119118 (27.4%)	120255 (27.6%)	120864 (27.8%)	123269 (28.0%)	126923 (28.4%)	128930 (28.7%)	131108 (29.2%)	133349 (29.7%)	136017 (30.2%)	137961 (30.7%)	
Bipolar disorders alone, n (%)		120590 (27.7%)	123159 (28.3%)	124495 (28.7%)	129880 (29.6%)	138032 (30.9%)	142118 (31.7%)	144788 (32.3%)	147032 (32.8%)	150664 (33.5%)	153695 (34.2%)	267311 (32.0%)
Schizophrenia spectrum disorders alone, n (%)		247899 (57.0%)	247771 (56.9%)	247784 (57.1%)	249501 (56.7%)	250380 (56.1%)	251902 (56.1%)	252608 (56.3%)	253031 (56.4%)	253966 (56.4%)	254192 (56.5%)	450670 (53.9%)
Both BD and SZ, n (%)		66230 (15.2%)	64299 (14.8%)	61878 (14.3%)	60143 (13.7%)	58168 (13.0%)	54705 (12.2%)	51495 (11.5%)	48720 (10.9%)	45506 (10.1%)	42068 (9.3%)	118621 (14.2%)

### Number and dose of psychotropic drugs

**Figure 2.**
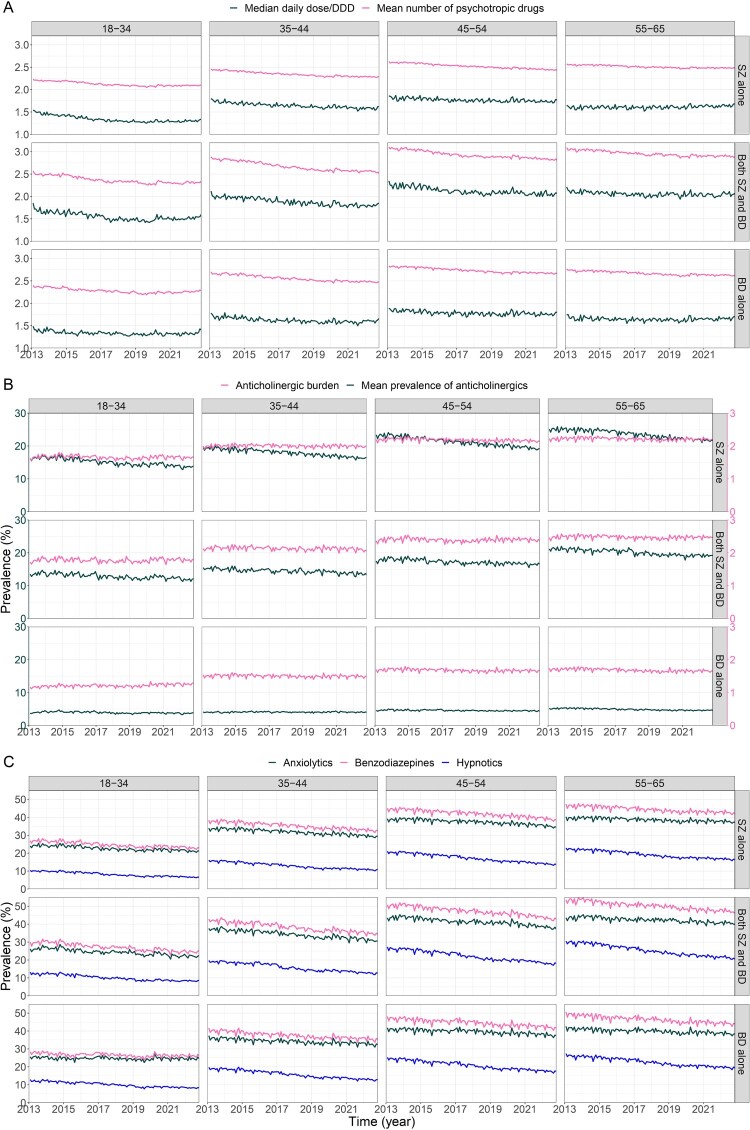
Changes in prescription practices for drugs with putative cognitive adverse effects in adults with schizophrenia, bipolar disorders or both, across age groups, for the period from 2013 to 2022. A: Mean monthly number and median total dose/DDD for psychotropic drugs. B: Mean monthly anticholinergic burden of psychotropic drugs and monthly prevalence of anticholinergic agent use. C: Monthly prevalence of benzodiazepine, anxiolytic and hypnotic drug use.

In 2013, treated adults with BD alone were prescribed a mean of 2.73 (median = 3) psychotropic drugs, those with SZ alone received 2.47 (median = 2) psychotropic drugs, and adults with both SZ and BD received 2.90 (median = 3) psychotropic drugs. In 2022, the mean number of psychotropic drugs per patient was 2.58 (median = 2) in adults with BD alone, 2.36 (median = 2) in adults with SZ alone, and 2.71 (median = 3) in adults with both conditions. The mean number of psychotropic drugs per patient significantly decreased across age groups and diagnoses between 2013 and 2022, except for adults with BD younger than 34 (Figure 2(A) & Table 2). The slowest decline was observed in adults aged 55–65 years.

**Table 2.** Time trends of prescription practices for drugs with putative cognitive side effects between 2013 and 2022 (*n* = 836,602). The mean annual variation of the mean number of psychotropic drugs, the median total daily dose of psychotropic drugs, the mean anticholinergic burden of psychotropic drugs, the frequency of receiving benzodiazepines and the frequency of receiving anticholinergic drugs were estimated by a mixed linear regression model including a random intercept and slope for each individual.

Variable		Number of psychotropic drugs		Total daily dose of psychotropic drugs	
Diagnosis group	Age group (years)	Mean annual variation (95% CI)	*p*-value	Mean annual variation (95% CI)	*p*-value
BD alone	18–34	0 (−0.002: 0.002)	0.703	**0.004 (0.001: 0.007)**	**0.016**
	35–44	**-0.018 (**−**0.002:** −**0.016)**	**<.001**	−**0.012 (**−**0.016:** −**0.008)**	**<.001**
	45–54	−**0.016 (**−**0.018:** −**0.014)**	**<.001**	−**0.010 (**−**0.014:** −**0.006)**	**<.001**
	55–65	−**0.011 (**−**0.013:** −**0.009)**	**<.001**	−**0.003 (**−**0.006:** −**0.001)**	**.036**
Both BD and SZ	18–34	−**0.014 (**−**0.017:** −**0.011)**	**<.001**	−**0.008 (**−**0.012:** −**0.004)**	**<.001**
	35–44	−**0.031 (**−**0.035:** −**0.027)**	**<.001**	−**0.029 (**−**0.035:** −**0.023)**	**<.001**
	45–54	−**0.024 (**−**0.028:** −**0.020)**	**<.001**	−**0.017 (**−**0.023:** −**0.011)**	**.002**
	55–65	−**0.014 (**−**0.018:** −**0.01)**	**<.001**	−**0.008 (**−**0.012:** −**0.004)**	**<.001**
SZ alone	18–34	−**0.010 (**−**0.011:** −**0.009)**	**<.001**	−**0.010 (**−**0.012:** −**0.008)**	**<.001**
	35–44	−**0.016 (**−**0.017:** −**0.015)**	**<.001**	−**0.015 (**−**0.017:** −**0.013)**	**<.001**
	45–54	−**0.016 (**−**0.017:** −**0.015)**	**<.001**	−**0.010 (**−**0.012:** −**0.008)**	**<.001**
	55–65	−**0.006 (**−**0.007:** −**0.005)**	**<.001**	**0.008 (0.006: 0.01)**	**<.001**
Variable		Frequency of anticholinergic agent use		Anticholinergic burden	
Diagnosis group	Age group (years)	Annual variation (95% CI)	*p*-value	Annual variation (95% CI)	*p*-value
BD alone	18–34	0.01% (−0.01%: 0.03%)	.544	**0.009 (0.007: 0.011)**	**<.001**
	35–44	**0.05% (0.01%: 0.09%)**	**.005**	−**0.005 (**−**0.008:** −**0.002)**	**<.001**
	45–54	**0.05% (0.01%: 0.09%)**	**.006**	−**0.004 (**−**0.007:** −**0.001)**	**<.001**
	55–65	0 (−0.02%: 0.02%)	.835	−**0.016 (**−**0.019:** −**0.013)**	**<.001**
Both BD and SZ	18–34	−**0.13% (**−**0.19%:** −**0.07%)**	**<.001**	0.004 (0: 0.008)	.050
	35–44	−**0.06% (**−**0.16%: 0.04%)**	**.151**	−0.002 (−0.007: 0.003)	.272
	45–54	−**0.18% (**−**0.26%:** −**0.10%)**	**<.001**	0 (−0.004: 0.004)	.851
	55–65	−**0.29% (**−**0.37%:** −**0.21%)**	**<.001**	−**0.005 (**−**0.009:** −**0.001)**	**.001**
SZ alone	18–34	−**0.28% (**−**0.32%:** −**0.24%)**	**<.001**	−0.002 (−0.004: 0)	.075
	35–44	−**0.30% (**−**0.34%:** −**0.62%)**	**<.001**	0 (−0.002: 0.002)	.174
	45–54	−**0.37% (**−**0.41%:** −**0.33%)**	**<.001**	−0.004 (−0.006: −0.002)	.005
	55–65	−**0.33% (**−**0.37%:** −**0.29%)**	**<.001**	0 (−0.002: 0.002)	.583
Variable		Frequency of benzodiazepine use			
Diagnosis group	Age group (years)	Annual variation (95% CI)	*p*-value		
BD alone	18–34	−**0.26% (**−**0.32%:** −**0.20%)**	**<.001**		
	35–44	−**0.62% (**−**0.70%:** −**0.54%)**	**<.001**		
	45–54	−**0.61% (**−**0.69%:** −**0.53%)**	**<.001**		
	55–65	−**0.52% (**−**0.60%:** −**0.44%)**	**<.001**		
Both BD and SZ	18–34	−**0.66% (**−**0.74%:** −**0.58%)**	**<.001**		
	35–44	−**0.88% (**−**1.00%:** −**0.76%)**	**<.001**		
	45–54	−**0.81% (**−**0.91%:** −**0.71%)**	**<.001**		
	55–65	−**0.72% (**−**0.82%:** −**0.62%)**	**<.001**		
SZ alone	18–34	−**0.48% (**−**0.52%:** −**0.44%)**	**<.001**		
	35–44	−**0.63% (**−**0.69%:** −**0.57%)**	**<.001**		
	45–54	−**0.66% (**−**0.72%:** −**0.6%)**	**<.001**		
	55–65	−**0.50% (**−**0.56%:** −**0.44%)**	**<.001**		

**Figure 3.**
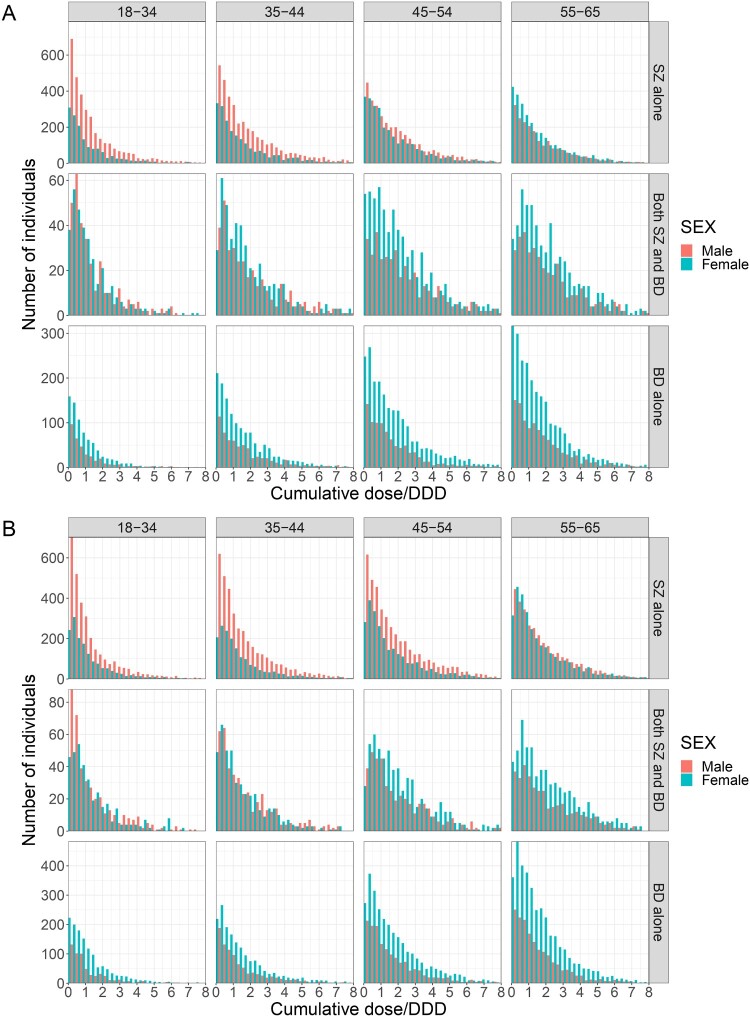
Total daily dose/DDD of psychotropic medications across sex, diagnoses and age groups in 2013 (A) and in 2022 (B).

Total daily dose followed a left-skewed distribution (Figure 3). We therefore reported the median value. In 2013, the median total daily dose/DDD ratio for psychotropic drugs was 1.7 for BD alone, 1.66 for SZ alone, and 2 for individuals with both diagnoses. In 2022, it was 1.68 for BD alone, 1.62 for SZ alone, and 1.95 for individuals with both diagnoses. The total daily dose significantly increased for adults with BD younger than 34 and adults with SZ aged between 55 and 65 years old, but decreased significantly over time in other groups (Figure 2(A) & Table 2).

**Figure 4.**
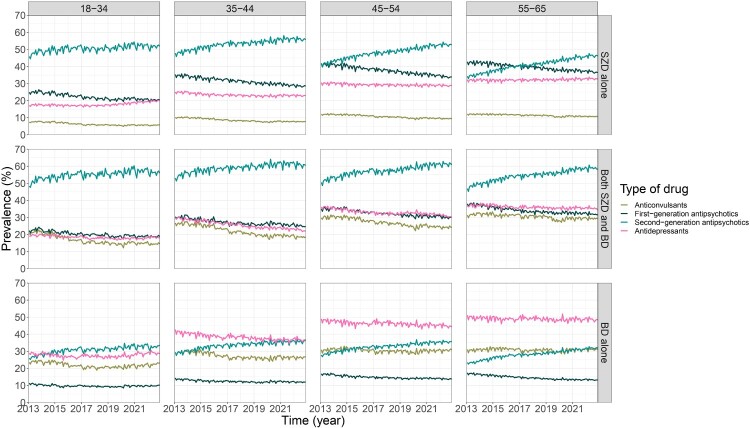
Monthly prevalence of the delivery of first- and second-generation antipsychotics, antidepressants and anticonvulsants in adults with schizophrenia, bipolar disorders or both, from 2013 to 2022.

The number of deliveries of first-generation antipsychotics, antidepressants and anticonvulsants decreased in adults with SZ and BD, accounting for the decrease in the number of psychotropic drugs (Figure 4). However, the number of deliveries of second-generation antipsychotics increased in most age groups, contrasting with the observed trend towards deprescription. Interestingly, the decrease in the number of psychotropic drugs stopped after 2020 across most age groups and diagnoses, and even increased in adults under 34 (Table 3). Besides, the total dose of psychotropic drugs globally increased after 2020.

**Table 3.** Time trends of prescription practices for drugs with putative cognitive side effects between 2013 and 2019 and between 2020 and 2022 to account for the COVID-19 pandemic (***n*** = 836,602). The annual variations, i.e. the slopes, were estimated with a mixed segmented linear regression model.

Variable		Number of psychotropic drugs				Total daily dose of psychotropic drugs			
Diagnosis group	Age group (years)	Annual variation before 2020	*p*-value	Annual variation after 2020	*p*-value	Annual variation before 2020	*p*-value	Annual variation after 2020	*p*-value
BD alone	18–34	−0.001 (−0.003: 0.001)	.262	**0.028 (0.006: 0.050)**	**.008**	0 (0.0018)	0.980	**0.059 (0.015)**	**<.001**
	35–44	−**0.018 (**−**0.021:** −**0.015)**	**<.001**	−0.005 (−0.033: 0.023)	.162	−**0.015 (.0024)**	**<.001**	**0.045 (.020)**	**<.001**
	45–54	−**0.015 (**−**0.018:** −**0.012)**	**<.001**	−0.014 (−0.040: 0.012)	.001	−**0.012 (.0022)**	**<.001**	**0.027 (.018)**	**<.001**
	55–65	−**0.010 (**−**0.013:** −**0.007)**	**<.001**	−0.002 (−0.026: 0.022)	.015	−**0.006 (.0021)**	**.009**	**0.041 (.018)**	**<.001**
Both BD and SZ	18–34	−**0.017 (**−**0.02:** −**0.014)**	**<.001**	**0.036 (0.002: 0.070)**	**.031**	−**0.012 (.0026)**	**<.001**	**0.086 (.026)**	**.001**
	35–44	−**0.032 (**−**0.036:** −**0.028)**	**<.001**	−0.023 (−0.069: 0.023)	.562	−**0.030 (.0035)**	**<.001**	**0.006 (.036)**	**.026**
	45–54	−**0.023 (**−**0.027:** −**0.019)**	**.003**	−0.043 (−0.087: 0.001)	.062	−**0.012 (.0034)**	**<.001**	**0.008 (.034)**	**.023**
	55–65	−**0.017 (**−**0.021:** −**0.013)**	**<.001**	−0.014 (−0.058: 0.03)	.241	−**0.004 (.0034)**	**.027**	**0.044 (.034)**	**.001**
SZ alone	18–34	−**0.011 (.0007)**	**<.001**	**0.008 (.006)**	**.204**	−**0.014 (.0011)**	**<.001**	**0.056 (.009)**	**<.001**
	35–44	−**0.016 (.0010)**	**<.001**	−**0.018 (.008)**	**.003**	−**0.014 (.0015)**	**<.001**	**0.019 (.012)**	**.006**
	45–54	−**0.016 (.0009)**	**<.001**	−**0.034 (.008)**	**<.001**	−**0.011 (.0014)**	**.014**	**0.007 (.013)**	**<.001**
	55–65	−**0.005 (.0010)**	**<.001**	−0.004 (.008)	.119	**0.006 (.0014)**	**<.001**	**0.056 (.013)**	**<.001**
Variable		Prevalence of anticholinergic agent use				Anticholinergic burden			
Diagnosis group		Annual variation before 2020	*p*-value	Annual variation after 2020	*p-*value	Annual variation before 2020	*p*-value	Annual variation after 2020	*p*-value
BD alone	18–34	0.01% (.0001)	.405	−0.07% (.13%)	.596	0.004 (.0021)	.120	0.002 (.021)	.070
	35–44	**0.05% (.02%)**	**.025**	0.01% (.18%)	.629	−**0.006 (.0032)**	**.001**	0.006 (.027)	.878
	45–54	**0.05% (.02%)**	**.032**	0.02% (.17%)	.582	−**0.007 (.0029)**	**<.001**	−0.014 (.025)	.504
	55–65	0 (.01%)	.630	0.08 (.16%)	.224	−**0.009 (.0029)**	**<.001**	0.006 (.024)	.861
Both BD and SZ	18–34	−**0.12% (.04%)**	**.003**	−0.31% (.03%)	.421	0 (.0036)	.880	0.011 (.036)	.767
	35–44	−**0.04% (.05%)**	**.003**	−0.52% (.05%)	.697	−0.006 (.0049)	.219	−0.009 (.050)	.160
	45–54	−**0.18% (.05%)**	**.003**	−0.24% (.05%)	.887	−0.003 (.0047)	.589	−0.005 (.048)	.172
	55–65	−**0.30% (.05%)**	**<.001**	−0.13% (.05%)	.720	−0.010 (.0047)	.031	−0.011 (.048)	.577
SZ alone	18–34	−**0.27% (.02%)**	**<.001**	−**0.52% (.17%)**	**.003**	−**0.005 (.0014)**	**<.001**	0 (.013)	.972
	35–44	−**0.27% (.03%)**	**<.001**	−**0.61% (.24%)**	**.002**	−**0.003 (.0020)**	**<.001**	−0.027 (.018)	.145
	45–54	−**0.36% (.03%)**	**<.001**	−**0.59% (.23%)**	**.003**	−**0.008 (.0019)**	**.004**	−0.042 (.018)	.016
	55–65	−**0.33% (.03%)**	**.029**	−**0.52% (.23%)**	**.003**	−**0.005 (.0020)**	**<.001**	0.011 (.018)	.507
Variable		Prevalence of benzodiazepine use							
Diagnosis group		Annual variation before 2020	*p*-value	Annual variation after 2020	*p*-value				
BD alone	18–34	−**0.28% (.04%)**	**<.001**	−0.09% (.31%)	.780				
	35–44	−**0.63% (.05%)**	**<.001**	−0.68% (.41%)	.148				
	45–54	−**0.58% (.05%)**	**<.001**	−**1.16% (.37%)**	**.004**				
	55–65	−**0.52% (.04%)**	**<.001**	−0.64% (.36%)	.129				
Both BD and SZ	18–34	−**0.68% (.05%)**	**<.001**	−0.17% (.48%)	<.001				
	35–44	−**0.86% (.07%)**	**.005**	−1.14% (.66%)	.141				
	45–54	−**0.75% (.07%)**	**<.001**	−**1.94% (.63%)**	**.005**				
	55–65	−**0.71% (.07%)**	**<.001**	−0.84% (.63%)	.291				
SZ alone	18–34	−**0.48% (.02%)**	**<.001**	−**0.52% (.20%)**	**.009**				
	35–44	−**0.62% (.03%)**	**<.001**	−**0.52% (.27%)**	**.018**				
	45–54	−**0.61% (.03%)**	**<.001**	−**1.58% (.26%)**	**<.001**				
	55–65	−**0.48% (.03%)**	**<.001**	−**0.52% (.27%)**	**.007**				

### Anticholinergic agents and burden

In 2013, 9,387 (7.8%) adults with BD alone, 77,713 (31.3%) with SZ alone, and 19,038 (28.7%) with both conditions used anticholinergic agents. In 2022, 11,870 (7.7%) adults with BD alone, 68,696 (27.0%) with SZ alone, and 11,034 (26.2%) with both conditions used anticholinergic agents. The frequency of anticholinergic agents decreased in all adults with SZ (Figure 2(B) & Table 2). It decreased the slowest for the 18–34 age group. In adults with BD, the frequency of anticholinergic agents increased in those aged 35–54 years and remained stable in other age groups. When looking specifically after 2020, the frequency of anticholinergic agent use was stable in adults with BD alone but kept decreasing in adults with SZ alone (Table 3).

In 2013, the mean cumulative anticholinergic burden was 1.60 ± 1.75 in adults with BD alone, 2 ± 2.14 in adults with SZ alone, and 2.24 ± 2.16 in adults with both conditions. In 2022, the mean cumulative anticholinergic burden was 1.58 ± 1.70 in adults with BD alone, 2.02 ± 2.12 in adults with SZ alone, and 2.23 ± 2.15 in adults with both conditions. Considering that cumulative anticholinergic burden ≥3 is high (Lisibach et al., 2022), 55,299 (45.9%) adults with BD and 128,223 (51.7%) adults with SZD had a high anticholinergic burden over the year 2013, while 64,776 (42.1%) adults with BD and 125,644 (49.4%) adults with SZD had a high anticholinergic burden due to psychotropic drugs over the year 2022. Anticholinergic burden due to psychotropics decreased significantly but very slightly over time in adults with BD over 35 (Table 2 & Figure 2(B)). In adults with SZ alone, anticholinergic burden was stable. When using another anticholinergic burden scale, the anticholinergic burden exhibited a global stability over time (Supplemental Material Fig. S1). Besides, the variations found with the Salahudeen's scale were non-significant in the subgroup analyses with CRIDECO Anticholinergic Load Scale (Supplemental Material Table S3), supporting the relative stability of anticholinergic burden. The cumulative anticholinergic burden decreased more significantly when considering non-psychotropic drugs (Supplemental Material Table S3).

### Benzodiazepines

In 2013, 89,557 (74.3%) adults with BD alone, 155,266 (62.6%) adults with SZ alone, and 47,530 (71.8%) adults with both SZ and BD used benzodiazepines over the course of the year. In 2022, 105,171 (68.4%) adults with BD alone, 144,128 (56.7%) with SZ alone, and 27,032 (64.3%) with both conditions used benzodiazepines. The frequency of benzodiazepine use decreased significantly in adults across age groups and diagnoses (Figure 2(C)). This decrease was slower in adults with BD alone under 34 than in other groups (Table 2). After 2020, benzodiazepine use continued to decline in adults with SZ alone but remained stable in those with BD alone (Table 3).

## Discussion

We aimed to evaluate recent trends in the prescription practices with putative adverse cognitive effects in adults with SZD and BD between 2013 and 2022. We reported a decrease in the number and total dose of psychotropic drugs and the frequency of benzodiazepine use between 2013 and 2022, suggesting a better consideration of cognitive and other adverse effects in this population. However, the use of anticholinergic agents and the anticholinergic burden did not consistently decrease, suggesting that prescribers could further consider reducing the anticholinergic burden.

The mean numbers of psychotropic drugs reported were similar to previous estimates for SZ (Heald et al., 2017; Nielsen et al., 2010) and BD (Singh et al., 2022). It globally decreased from 2013 to 2022, suggesting that prescribers became more cautious about psychotropic polypharmacy. This decrease may have lowered the risks of cognitive impairment, adverse effects, and healthcare costs while enhancing patient engagement. The number of psychotropic drugs remained stable in young adults with BD and declined the slowest in adults aged 54–65 years, suggesting that reducing polypharmacy should be promoted in those age groups, especially in older patients who are more sensitive to cognitive adverse effects (Loggia et al., 2019). Further studies are required to confirm that the decrease in the number of psychotropics did not lead to an increase in relapse rates. The decrease in the number of psychotropics stopped after 2020 in most age groups. This finding might be explained by an increase in depressive symptoms and stress during and after the COVID-19 pandemic, resulting in the prescription of additional treatments (Li et al., 2020). Alternatively, prescriptions may have been automatically renewed due to the disorganisation of health care during the pandemic, interrupting the deprescription trend. Our results emphasise the need for prescribers to re-consider deprescribing psychotropics now that the pandemic has ended to restore the initial trend.

The total daily dose in this study was lower than that reported in previous studies on BD (Lin et al., 2022) and SZ (Nielsen et al., 2010). It might be explained by the fact that they included inpatients and reported the mean rather than the median dose/DDD. From 2013 to 2022, the total daily dose decreased significantly except in young adults with BD and older adults with SZ. In older adults with SZ, the total daily dose of psychotropics increased while the number of psychotropics decreased. This result suggests that prescribers tended to avoid polypharmacy in this age group but compensated for this by increasing the doses of the remaining drugs. Patients’ total dose of psychotropics increased after 2020, underlining the need to encourage dose reduction again.

The annual prevalence of anticholinergic agent use reported here is higher than reported in other French cohorts (Vidal et al., 2023, 2024). Similarly, the annual prevalences of high anticholinergic burden in BD and SZD were higher than those reported before using Salahudeen's scale (Vidal et al., 2023, 2024) or the Anticholinergic Drug Scale (Eum et al., 2017, 2021). Annual prevalence is not directly comparable to the point-prevalence reported in those previous studies because the probability of having anticholinergic medications over a year is likely higher than over one visit in cross-sectional studies, but those are the only existing comparators. Altogether, those findings highlight that anticholinergic burden constitutes a public health concern in BD and SZ. The frequency of anticholinergic agent use decreased significantly in adults with SZ, as reported before in inpatient settings (Toto et al., 2019; Ying et al., 2023). This finding suggests a better consideration of adverse cognitive effects. By contrast, the anticholinergic burden remained globally constant between 2013 and 2022, suggesting a lack of awareness regarding the risks associated with high anticholinergic burden in clinical practice and highlighting the need for international guidelines on managing anticholinergic burden in BD and SZ. Although psychotropics with a high anticholinergic burden are sometimes necessary to treat symptoms and maintain remission in BD and SZD, other medications, including non-psychotropics, can also contribute to the cumulative anticholinergic burden. Anticholinergic burden scales can help identify the anticholinergic properties of those adjunctive medications. Moreover, prescriptions of anticholinergic agents are sometimes automatically renewed, despite the WHO's recommendation to prescribe them only to individuals with significant extrapyramidal side-effects when dose reduction and switching strategies have proven ineffective, preferably over short-term periods (WHO, 2019).

The prevalence of benzodiazepine use in SZ and BD patients was higher than previous estimates (Haro et al., 2006). To note, France has one of the highest rates of benzodiazepine prescription in Europe (Boyd et al., 2015). We observed a significant decrease in benzodiazepine use between 2013 and 2022, contrasting with the increase reported in Sweden and Finland in recent decades (Taipale et al., 2021). The decreasing use of benzodiazepines is consistent with findings for the French general population (Bénard-Laribière et al., 2017) and recent international guidelines in SZ and BD (National Institute for Health and Clinical Excellence., 2014; Yatham et al., 2018). Besides, this decline continued after 2020 in adults with SZ but stopped in adults with BD. This suggests that people with SZ were less affected by anxiety during and after the pandemic than people with BD. To note, in 2012 in France the prescription of clonazepam was restricted to neurologists,^1^ potentially contributing to the reduction in benzodiazepine use in BD and SZD over the study period. Prescribing benzodiazepines is only appropriate to treat anxiety over short-term periods, after which benzodiazepines should be gradually discontinued to prevent withdrawal or rebound anxiety (WHO, 2023; Yatham et al., 2018). Our findings suggest that such guidelines successfully raised awareness of the hazards of benzodiazepine prescription.

Interestingly, many individuals were classified as having both SZ and BD during the study period, although those diagnoses are mutually exclusive. This result is consistent with previous findings in longitudinal data: 7.7% patients are diagnosed with BD within a year after an initial diagnosis of SZ or vice versa (Broder et al., 2018). These individuals were prescribed more psychotropic drugs at higher doses and had a higher anticholinergic burden, resulting in a higher risk of iatrogenic cognitive impairment. Alternate diagnoses of SZ and BD may be associated with greater instability in psychiatric follow-up, with frequent changes of psychiatrist and place of care, associated with more severe symptoms and more treatments. The alternation of diagnoses calls into question the reliability of the diagnosis of mental disorders in claims databases and the discriminant validity of the criteria defined to detect patients with those diagnoses.

The limitations of our study include a lack of explanation for the observed trends, which may have been influenced by factors such as international treatment guidelines, clinician experience, local prescribing habits, and available treatments (Dong et al., 2019). Our analysis focused on outpatient settings, so the trends observed may not apply to inpatients. Data for adherence to treatment were not available and we therefore assumed that all the prescribed treatments were both delivered and used. However, patients with SZ or BD have low-to-moderate levels of treatment adherence (Stephenson et al., 2012). The delivered dose/DDD definition did not account for treatments with narrow therapeutic windows, like lithium, for which dosing is based on serum lithium concentration. We did not consider the duration of the illness as it was not possible to determine reliably the date of onset of the mental disorder, due to the lack of data relating to psychiatric hospitalisations before 2007. We partially addressed this issue by stratifying our sample for age, but future studies should consider the duration of illness more explicitly. We focused on prescribing practices that may lead to cognitive impairment in SZ and BD, but those practices are also associated with other adverse effects (e.g. metabolic syndrome, constipation). Deprescription or dose reduction of psychotropics, especially anticholinergic drugs, might reduce the risk of peripheral adverse reactions such as constipation or urinary retention, improving physical health and quality of life (Chengappa et al., 2025; Lupu et al., 2021).

## Conclusion

Overall, we detected trends towards reducing prescription practices with possible adverse cognitive effects in adults with SZ and BD in France over the last decade that have probably improved cognitive health. However, considering the high prevalence of those prescription practices in 2022, deprescription of anticholinergic medications should be promoted further in future clinical recommendations now that the COVID-19 pandemic is over. Additional interventions to improve cognitive function in SZ and BD patients, whether pharmacological or otherwise, should also be supported.

## Supplementary Material

Supplemental Material

## Data Availability

Access to the French National Health Data System (SNDS) database is available only for individuals with specific authorisation.

## Footnotes

1 https://archive.ansm.sante.fr/S-informer/Points-d-information-Points-d-information/Rivotril-R-clonazepam-Modification-des-conditions-de-prescription-et-de-delivrance-Point-d-information.
