# Supplementary material for: Nationwide trends in psychiatric prescription practices for drugs with putative cognitive adverse effects in schizophrenia spectrum and bipolar disorders from 2013 to 2022
Source: J Pharm Policy Pract. 2026 Mar 31;19(1):2650543. doi: 10.1080/20523211.2026.2650543 (PMC13040571; doi:10.1080/20523211.2026.2650543)
Supplement: Supplemental Material [file JPPP_A_2650543_SM8055.docx]

Supplementary Information

Nationwide trends in psychiatric prescription practices for drugs with putative cognitive adverse effects in schizophrenia spectrum and bipolar disorders from 2013 to 2022

Graphs were plotted with the R package *ggplot2* version 3.4.3 [(1)](https://www.zotero.org/google-docs/?BAvCkO).

**Supplementary material SM1: Delivery periods for two types of medication packaging**

We considered two types of packaging that could be delivered:

- Deliveries of drugs for periods of less than 28 days (estimated to last 14 days on average) (with 5 to 15 pills or injectable forms, such as fluphenazine and risperidone depot injections). We estimated the time between deliveries at 7 to 21 days (7-day grace period). If no follow-up delivery was recorded within 21 days of the initial delivery or if no repeat delivery of the drug occurred before December 31st, 2022, we considered the dose missing. We avoided inflated dose values due to patients stockpiling medication by considering the treatment to be taken at the mean dose over 14 days for intervals of less than seven days between deliveries.
- Deliveries of drugs for a period of 28 to 30 days (with more than 20 pills). French pharmacists are not allowed to provide more than 30 days of treatment at a time (according to article R. 5132-12 of the French Public Health Code^^[[1]](#footnote-0)^^). We considered the interval between deliveries to range from 21 to 35 days (7-day grace period). If no subsequent delivery was recorded within 35 days of the initial delivery, if the delivery was recorded in December 2022 or if no repeat delivery of the drug occurred before December 31st, 2022, we considered the dose missing. We avoided artificially high dose values due to patients stockpiling medication by considering the treatment to be taken at the mean dose for 28 days if the interval between deliveries was less than 21 days.

We did not use a strict definition of treatment duration, which would assume that treatment stops 28 days after dispensing 28 tablets. This approach is not suitable given the frequent interruptions in psychotropic use due to factors such as forgetfulness, adverse effects, or intermittent symptoms [(2)](https://www.zotero.org/google-docs/?mW1SBG).

**Supplementary material SM2: Accounting for the COVID-19 pandemic in the regression models**

We used segmented regression models [(3)](https://www.zotero.org/google-docs/?hw7H0L) to account for the impact of the COVID-19 pandemic on the consumption of psychotropic drugs [(4)](https://www.zotero.org/google-docs/?Fsf6Zq). Time was considered in two ways: time in years from 2013 to 2020 (time1 variable) and time in years after 2020 onward (time2 variable).

Let *y* be the year.

- If *y* ≤ 2020, time1 = *y* - 2013 and time2 = 0.
- If *y* > 2020, time1 = 7 (= 2020 - 2013) and time2 = *y* - 2020.

The definitions of time1 and time2 were established such that for any value of *y*, the equation time1 + time2 = *y* holds true [(3)](https://www.zotero.org/google-docs/?z58wnF).

We also included a binary variable set to 1 for the year 2020 and onwards, and to 0 for the years before 2020, to account for the change in the intercept in 2020 [(4)](https://www.zotero.org/google-docs/?JYshCI).

**Figure S1. Mean monthly anticholinergic burden computed by two different anticholinergic burden scales (Salahudeen’s scale and CRIDECO ALS) when considering only psychotropic drugs or all prescribed drugs in adults with schizophrenia, bipolar disorders or both, across age groups, for the period from 2013 to 2022.**


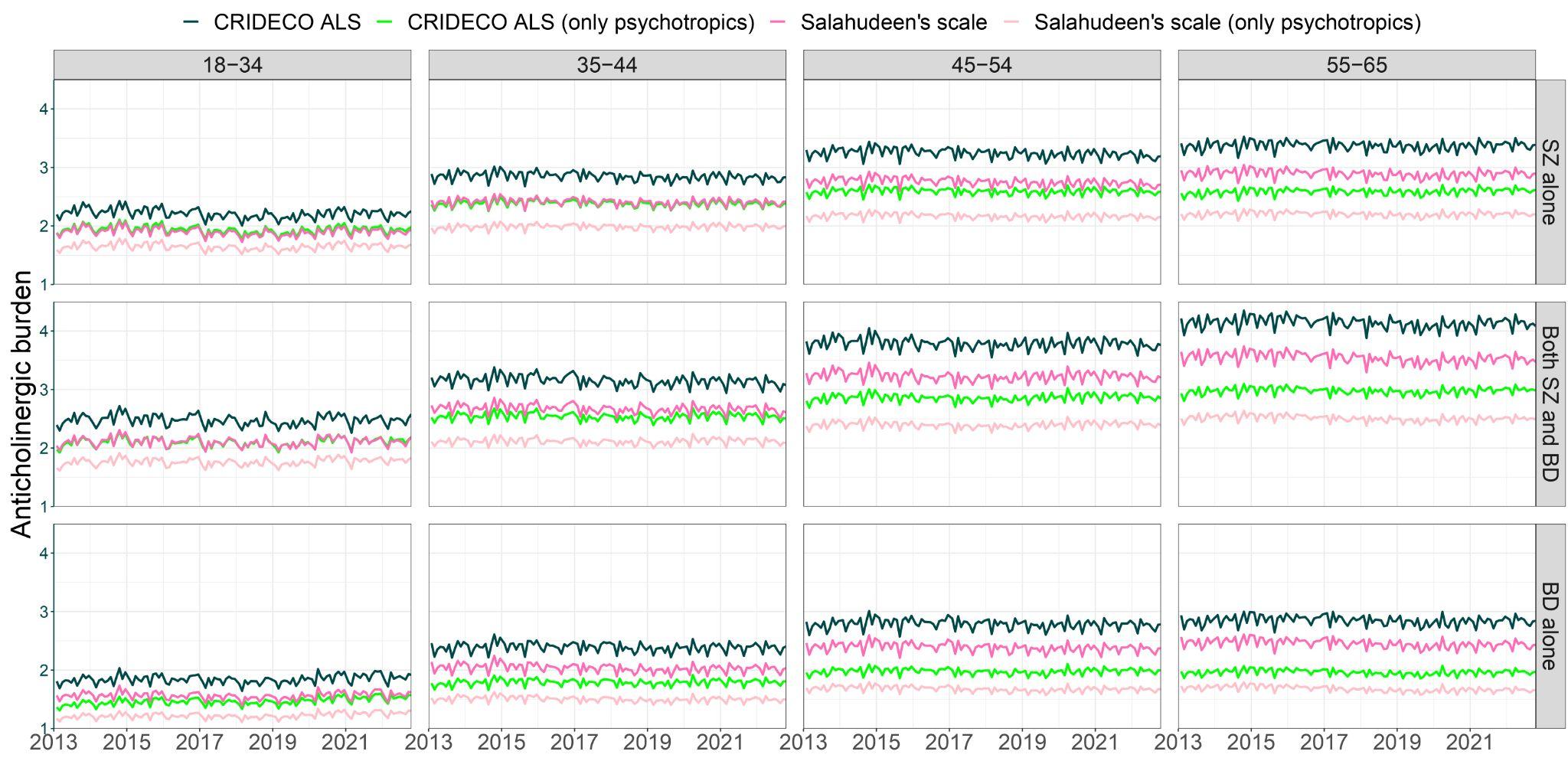


**Table S1. ICD-10 diagnosis codes used in the study**

| Disorders | ICD-10 code | Description |
| --- | --- | --- |
| Schizophrenia spectrum disorders | F20.0 | Paranoid schizophrenia |
|  | F20.1 | Hebephrenic schizophrenia |
|  | F20.2 | Catatonic schizophrenia |
|  | F20.3 | Undifferentiated schizophrenia |
|  | F20.4 | Post-schizophrenic depression |
|  | F20.5 | Residual schizophrenia |
|  | F20.6 | Simple schizophrenia |
|  | F20.8 | Other schizophrenia |
|  | F20.9 | Schizophrenia, unspecified |
|  | F21 | Schizotypal disorder |
|  | F22 | Persistent delusional disorders |
|  | F23 | Acute and transient psychotic disorders |
|  | F24 | Shared psychotic disorder |
|  | F25 | Schizoaffective disorders |
|  | F28 | Other nonorganic psychotic disorders |
|  | F29 | Unspecified nonorganic psychosis |
| Bipolar disorders | F30 | Manic episode |
|  | F31 | Bipolar disorders |

**Table S2. Definition of the exclusion criteria**

| Comorbid conditions | | Definition period | ICD-10 codes and/or medical procedures and/or treatments |
| --- | --- | --- | --- |
| Mental retardation | | Over the study period | F7 |
| Neurological comorbid conditions | Traumatic brain injury | From initial diagnosis to the end of the study period | S020, S021, S028, S0291, S0402, S0403, S0404, S06, S071, T744 [(5)](https://www.zotero.org/google-docs/?zkQVtc) |
|  | Parkinson’s disease |  | G20, F023 [(6)](https://www.zotero.org/google-docs/?4p9Qar) |
|  | Dementia |  | F00-F03, F051, G30, G311 [(7)](https://www.zotero.org/google-docs/?ot4v2i)  **AND/OR**  At least 3 reimbursements of anti-Alzheimer drugs: ATC codes starting with NO6DA, and N06DX01 [(6)](https://www.zotero.org/google-docs/?Gly3nT) |
|  | Multiple sclerosis |  | G35 [(6)](https://www.zotero.org/google-docs/?fPsr5L) |
|  | Epilepsy |  | G40, G41 [(6)](https://www.zotero.org/google-docs/?W56sqn) |
|  | Cerebrovascular disease |  | I67-69 |
| ECT | | In the preceding year | Procedure (CCAM code): Session of electroconvulsive therapy (AZRP001) |

*Notes. CCAM: Common Classification of Medical Acts. A complete list of existing CCAM codes can be found at https://assurance-maladie.ameli.fr/etudes-et-donnees/actes-techniques-ccam.*

**Table S3. Mean annual variations between 2013 and 2022 in anticholinergic burden computed by the CRIDECO ALS or by Salahudeen’s scale for all drugs or only psychotropics (n = 836,602).** The annual variations, i.e. the slopes, of the mean anticholinergic burden per patient were estimated by a linear regression model.

| Diagnosis group | Age group (years) | **Anticholinergic burden measured by Salahudeen’s scale** | | | | **Anticholinergic burden measured by CRIDECO ALS** | | | |
| --- | --- | --- | --- | --- | --- | --- | --- | --- | --- |
|  |  | **only psychotropic drugs** | | **all medications** | | **only psychotropic drugs** | | **all medications** | |
|  |  | Annual variation (95% CI) | *p*-value | Annual variation (95% CI) | *p*-value | Annual variation (95% CI) | *p*-value | Annual variation (95% CI) | *p*-value |
| BD alone | 18-34 | **0.009 (0.007: 0.011)** | **<.001** | 0.004 (0: 0.008) | .070 | **0.014 (0.012: 0.016)** | **<.001** | **0.008 (0.006: 0.01)** | **<.001** |
|  | 35-44 | **-0.005 (-0.008: -0.002)** | **<.001** | **-0.006 (-0.011: -0.001)** | **<.001** | 0.001 (-0.002: 0.004) | .412 | -0.002 (-0.008: 0.004) | .712 |
|  | 45-54 | **-0.004 (-0.007: -0.001)** | **<.001** | **-0.007 (-0.012: -0.002)** | **<.001** | 0 (-0.003: 0.003) | .798 | -0.004 (-0.010: 0.002) | .594 |
|  | 55-65 | **-0.016 (-0.019: -0.013)** | **<.001** | **-0.008 (-0.012: -0.004)** | **<.001** | -0.001 (-0.004: 0.002) | .587 | -0.003 (-0.009: 0.003) | .665 |
| Both BD and SZ | 18-34 | 0.004 (0: 0.008) | .050 | 0 (-0.003: 0.003) | .997 | 0.003 (-0.001: 0.007) | .121 | -0.001 (-0.007: 0.005) | .807 |
|  | 35-44 | -0.002 (-0.007: 0.003) | .272 | **-0.009 (-0.017: -0.001)** | **.033** | -0.003 (-0.009: 0.003) | .279 | **-0.011 (-0.021: -0.001)** | **.022** |
|  | 45-54 | 0 (-0.004: 0.004) | .851 | -0.005 (-0.013: 0.003) | .185 | 0.002 (-0.004: 0.008) | .685 | -0.005 (-0.015: 0.005) | .253 |
|  | 55-65 | **-0.005 (-0.009: -0.001)** | **.001** | **-0.011 (-0.019: -0.003)** | **.008** | 0.001 (-0.005: 0.007) | .854 | -0.005 (-0.015: 0.005) | .248 |
| SZ alone | 18-34 | -0.002 (-0.004: 0) | .075 | **-0.005 (-0.007: -0.003)** | **<.001** | **-0.003 (-0.005: -0.001)** | **<.001** | **-0.007 (-0.009: -0.005)** | **<.001** |
|  | 35-44 | 0 (-0.002: 0.002) | .174 | **-0.005 (-0.007: -0.003)** | **<.001** | -0.002 (-0.004: 0) | .074 | **-0.007 (-0.009: -0.005)** | **<.001** |
|  | 45-54 | **-0.004 (-0.006: -0.002)** | **.005** | **-0.010 (-0.013: -0.007)** | **.004** | -0.001 (-0.003: 0.002) | .550 | **-0.002 (-0.004: 0.002)** | **<.001** |
|  | 55-65 | 0 (-0.002: 0.002) | .583 | **-0.005 (-0.007: -0.003)** | **<.001** | **0.005 (0.003: 0.007)** | **<.001** | 0 (-0.002: 0.002) | .583 |

References

[1. Wickham H. ggplot2: Elegant Graphics for Data Analysis [Internet]. Springer-Verlag New York. Vol. 978-3-319-24277‑4. ISBN; 2016. Disponible sur: https://ggplot2.tidyverse.org](https://www.zotero.org/google-docs/?8Wpuvo)

[2. Stephenson JJ, Tunceli O, Gu T, Eisenberg D, Panish J, Crivera C, et al. Adherence to oral second-generation antipsychotic medications in patients with schizophrenia and bipolar disorder: physicians’ perceptions of adherence vs.  pharmacy claims. Int J Clin Pract. juin 2012;66(6):565‑73.](https://www.zotero.org/google-docs/?8Wpuvo)

[3. Wagner AK, Soumerai SB, Zhang F, Ross-Degnan D. Segmented regression analysis of interrupted time series studies in medication use research. J Clin Pharm Ther. août 2002;27(4):299‑309.](https://www.zotero.org/google-docs/?8Wpuvo)

[4. Benistand P, Vorilhon P, Laporte C, Bouillon-Minois JB, Brousse G, Bagheri R, et al. Effect of the COVID-19 pandemic on the psychotropic drug consumption. Front Psychiatry. 2022;13:1020023.](https://www.zotero.org/google-docs/?8Wpuvo)

[5. Hedegaard H, Johnson RL, Warner M, Chen LH, Annest JL. Proposed Framework for Presenting Injury Data Using the International Classification of Diseases, Tenth Revision, Clinical Modification (ICD-10-CM)  Diagnosis Codes. Natl Health Stat Rep. 22 janv 2016;(89):1‑20.](https://www.zotero.org/google-docs/?8Wpuvo)

[6. Quantin C, Roussot A, Cottenet J, Besson J. Méthode de la cartographie des pathologies et des dépenses de l’Assurance Maladie. 2015; Disponible sur: https://www.assurance-maladie.ameli.fr/etudes-et-donnees/par-theme/pathologies/cartographie-assurance-maladie/methode-cartographie-pathologies-depenses-assurance-maladie#text_155397](https://www.zotero.org/google-docs/?8Wpuvo)

[7. Bannay A, Chaignot C, Blotière PO, Basson M, Weill A, Ricordeau P, et al. The Best Use of the Charlson Comorbidity Index With Electronic Health Care Database to Predict Mortality. Med Care. févr 2016;54(2):188‑94.](https://www.zotero.org/google-docs/?8Wpuvo)

1. https://www.legifrance.gouv.fr/codes/article_lc/LEGIARTI000006915549 [↑](#footnote-ref-0)
